# Evaluation of the Application Value of Cottonseed Protein Concentrate as a Feed Protein Source in Broiler Chickens

**DOI:** 10.3390/ani13233706

**Published:** 2023-11-29

**Authors:** Xing Chen, Manqi Zhao, Aijuan Zheng, Adanan Purba, Zhimin Chen, Kai Qiu, Zedong Wang, Wenhuan Chang, Huiyi Cai, Guohua Liu

**Affiliations:** Key Laboratory for Feed Biotechnology of the Ministry of Agriculture and Rural Affairs, Institute of Feed Research, Chinese Academy of Agricultural Sciences, Beijing 100081, China; cx18222052919@163.com (X.C.); zhaomanqi2021@163.com (M.Z.); mhdadananpurba@usu.ac.id (A.P.); chenzhimin@caas.cn (Z.C.); qiukai@caas.cn (K.Q.); wangzedong@caas.cn (Z.W.); changwenhuan@caas.cn (W.C.); caihuiyi@caas.cn (H.C.); liuguohua@caas.cn (G.L.)

**Keywords:** cottonseed protein concentrate, growth performance, antioxidant capacity, immunoglobulin, broiler chicken

## Abstract

**Simple Summary:**

Cottonseed protein concentrate (CPC) is a novel plant protein source that has been developed using advanced cottonseed processing technology. It was extensively utilized as a replacement for fish meal in feed within the aquaculture industry. However, there was limited research on using CPC as a protein source to replace soybean meal in broiler diets. This study conducted a comprehensive analysis of the chemical and amino acid composition of CPC, and assessed its protein and amino acid digestibility, metabolizable energy value, and net energy value in the broiler chickens. Additionally, the experiment examined the effects of replacing 25% and 50% of soybean meal in feed with CPC on the growth performance, blood parameters, antioxidant capacity, and immunoglobulin content of broiler chickens. These findings provide valuable insights into the potential use of CPC as a substitute for soybean meal in broiler feed formulation.

**Abstract:**

Cottonseed protein concentrate (CPC) has the function of replacing soybean meal to maintain normal animal growth and development. This study involved 180 Arbor Acres (AA) broilers, which were randomly assigned to three different treatments. Each treatment had six replicates, with each replicate consisting of 10 chicks. The control group was fed a basal diet, while the CPC-1 and CPC-2 groups used CPC to replace 25% and 50% of the soybean meal in the basal diet, respectively. The study showed that replacing soybean meal with 25% CPC in broilers’ diets can maintain normal growth, while substituting 50% of soybean meal with CPC negatively affects the growth and development of broiler chickens. Furthermore, the CPC-1 group showed a significant increase in serum total antioxidant capacity, superoxide dismutase enzyme activity, and immunoglobulin content, along with a decrease in malondialdehyde content. Based on the research results mentioned above, it was speculated that CPC has the potential to replace around 25% of soybean meal in broiler feed without causing any negative impact on growth performance. This suggests that CPC could be a viable alternative to soybean meal in broiler diet.

## 1. Introduction

Soybean meal is a widely used protein source in poultry feed due to its high nutritional value and fairly balanced amino acid composition [1,2]. Regrettably, the escalating cost of soybean meal has imposed restrictions on its usage in animal feed, which posing a significant challenge to the advancement of animal husbandry [3]. Therefore, there is an urgent need to find alternative protein sources that can effectively replace soybean meal. Cottonseed meal is a byproduct of cotton production that has the advantage of large output and low production cost [4]. Cottonseed meal not only minimizes the waste of cottonseed resources, but it also contributes to the reduction of feed protein resources. However, because of the existence of some anti-nutritional substances, such as free gossypol (FG), it is necessary to process it in order to enhance its utilization efficiency [5].

The yield of cottonseed is remarkably high, while cottonseed protein concentrate (CPC) is a novel plant protein source that has been developed by enhancing cottonseed processing technology [6]. The preparation steps of CPC are as follows: cottonseed is physically separated by a shelling machine to obtain cottonseed, and the cottonseed embryo sheet formed after rolling is dephenolized via chemical reagents (a mixture of methanol and n-hexane solvent). The product is rapidly dried at low temperatures to produce the finished CPC [7]. This enables the removal of several anti-nutritional factors, including free gossypol, cyclopropene fatty acids, and mycotoxins, which had previously restricted the use of cottonseed meal [8]. Moreover, CPC shows a significant increase in the content of essential amino acids, particularly lysine, which is increased by over 40%. When compared to soybean meal, the amount of arginine and sulfur-containing amino acids (methionine + cysteine) is significantly increased. At present, CPC is widely used in the aquaculture industry as a substitute for fish meal in feed. A study conducted found that substituting 24% fish meal with CPC significantly increased the specific growth rate (SGR) of hybrid grouper [9]. Similarly, Wang et al. reported that replacing 15% and 30% fish meal with CPC resulted in an increased final body weight (FBW), weight gain rate (WGR), SGR, and protein efficiency ratio (PER) of litopenaeus vannamei [10].

However, limited research has been conducted on the application of CPC in broilers. The experiment examined the chemical composition, metabolic energy, and net energy of CPC, along with the content and digestibility of amino acids. The objective was to investigate the effects of CPC on the growth performance, blood parameters, antioxidant capacity, and immune function of broilers. The findings of this study can provide a theoretical basis for the application of CPC in broiler production.

## 2. Materials and Methods

### 2.1. Ethics Statement

The research was licensed by the ethical approval of the Animal Care and Use Committee of the Institute of Feed Research of the Chinese Academy of Agricultural Science (Statement No. AEC-CAAS-20191106, Beijing, China). The CPC was kindly provided by Xinjiang Jinlan Plant Protein Co., Ltd. (Shihezi, China).

### 2.2. Analysis of Chemical Composition, Energy, and Ileal Digestibility of CPC

#### 2.2.1. Chemical Composition

The CPC samples were crushed and passed through a 0.5 mm sieve, dried at 105 °C for four hours, and measured for dry matter (DM, AOAC #934.01). The samples were placed in a muffle furnace at 550 °C for 4 h to determine the ash. Crude protein (CP, AOAC #984.13), crude fiber (CF, AOAC #978.10), and crude fat (EE, AOAC #2003.05) content in CPC samples were analyzed according to AOAC International Standards. The amino acid composition of CPC sample in amino acid analyzer (Hitachi L-8900, Tokyo, Japan) was analyzed using ninhydrin post-column derivatization ion-exchange chromatography; oxidized methionine and cystine with high performance acid before analysis.

#### 2.2.2. Apparent Metabolizable Energy and Net Energy

The apparent metabolizable energy (AME) and net energy (NE) of broilers in CPC were determined in the calorimetric chambers. A total of 16 Arbor Acres broilers of comparable body weight aged 42 days old were randomly divided into two treatments (basal diet group and treatment diet group), each treatment with four replicates and two broilers per replicate. Broilers were randomly assigned to four respiratory chambers, and freely ate and drank to adapt to the new environment during the first 3 d. The composition of the experiment diet for determining the AME and NE of CPC in broilers were shown in Table 1. The O_2_ consumption and CO_2_ production were measured from the 3rd to the 7th day, which was used to calculate heat production. Air conditioners and heaters regulated the temperature and humidity in the calorimeter chamber. Gas was drawn out of the breathing chamber by a vacuum pump, and an analyzer measured the oxygen and carbon dioxide levels [11]. The collected excrement was concentrated in each compartment within five days and stored in the refrigerator. On the 7th day, broilers were weighed to calculate their weight gain. The sample was taken as standard benzoic acid, and the total energy was measured with an oxygen bomb calorimeter (IKA-C3000, Berlin, Germany) [12]. The Gross Energy intake value for CPC was determined using the following formula:

Gross Energy intake (GEI, MJ) = Gross Energy (GE, MJ/kg) × Feed intake (FI, kg).

Apparent metabolizable energy (AME) and excreta energy (E_EN_) were the total energy recovered from feed and manure, respectively, and Feed Intake (FI) was the feed intake of broilers. Nitrogen retention (RN) indicates the retention of nitrogen in the body. In addition, nitrogen intake (NI) and nitrogen efficiency (NE) represent dietary nitrogen uptake and excretion, respectively. Heat total production (HTP) values were estimated from the amount of carbon dioxide exhaled and the amount of oxygen consumed by the birds. AME, RN, AMEn, and RE values were determined using the following formulas [13]:

$$\mathrm{Heat}\;\mathrm{total}\;\mathrm{production}\;(\mathrm{HTP},\;\mathrm{KJ})=16.18\;\times \;{\mathrm{VO}}_{2}\;+\;5.02\;\times \;{\mathrm{VCO}}_{2};\;\mathrm{Retained}\;\mathrm{energy}\;(\mathrm{RE},\;\mathrm{KJ})=\mathrm{Apparent}\;\mathrm{metabolizable}\;\mathrm{energy}\;\mathrm{intake}\;(\mathrm{AMEI},\;\mathrm{KJ})\;-\;\mathrm{Heat}\;\mathrm{total}\;\mathrm{production}\;(\mathrm{HTP},\;\mathrm{KJ})\;\;\mathrm{Nitrogen}\;\mathrm{corrected}\;\mathrm{apparent}\;\mathrm{metabolizable}\;\mathrm{energy}\;(\mathrm{AMEn})=\mathrm{AME}\;-\;\mathrm{RN}$$

The Net energy (NE) values of feeds were calculated using the following equation:

Net energy (NE, MJ/kg)=(AMEI - HI) / FI;

This formula calculated the net energy value of raw materials:

Raw materials (NE)=Base diet (NE) - [Base diet (NE) - The diet to be tested (NE)] / A%,

where A is the proportion of raw materials tested in the diet.

#### 2.2.3. Ileal Digestibility

The apparent digestibility coefficient of dry matter, and the apparent and standard digestibility coefficient of protein and amino acid of CPC in broiler ileums, were calculated according to the proportion of titanium markers in the diet [14]. Table 2 shows the composition of test diets for the digestibility rate of dry matter, protein, and amino acid of CPC in the ileum of broilers. A total of 72 Arbor Acres broilers aged 42 days old were randomly divided into two treatments, each treatment with six replicates and six broilers per replicate. A configured nitrogen-free diet (NFD) was used to evaluate the basal endogenous loss (BEL) of amino acids, and TiO_2_ was used to determine the digestibility indicators. The ileal digestibility was calculated as follows:

$$\begin{array}{l} \mathrm{Apparent}\;\mathrm{dry}\;\mathrm{matter}\;\mathrm{digestibility}\;\mathrm{coefficient}\;(\mathrm{ADMD},\;\%)=[({\mathrm{Ti}}_{f}\;-\;{\mathrm{Ti}}_{d})/\;{\mathrm{Ti}}_{f}]\;\times \;100.\\ \mathrm{Apparent}\;\mathrm{protein}\;\mathrm{digestibility}\;\mathrm{coefficient}\;(\mathrm{APD},\;\%)=[100-({\mathrm{CP}}_{d}/{\mathrm{CP}}_{f})\;\times \;({\mathrm{Ti}}_{f}\;-\;{\mathrm{Ti}}_{d})]\;\times \;100. \end{array}$$

where the Ti_d_ and CP_d_ are the quantities of CP and TiO_2_ in the ileal digesta, respectively, and Ti_f_ and CP_f_ are the quantities of CP and TiO_2_ in the feed, respectively.

$$\mathrm{Apparent}\;\mathrm{ileal}\;\mathrm{digestibility}\;\mathrm{coefficient}\;(\mathrm{AID},\;\%)=[{\left(\mathrm{AA}/\mathrm{Ti}\right)}_{d}\;-{\left(\mathrm{AA}/\mathrm{Ti}\right)}_{f}]\;/\;{\left(\mathrm{AA}/\mathrm{Ti}\right)}_{d},$$

where (AA/Ti)_d_ and (AA/Ti)_f_ represent the proportion of amino acids in diet and chyme, respectively.

The basal endogenous amino acid (EAA) flow at the end of the ileal was calculated from the grams lost per kilogram of DMI. Td and Ti represent the content of Ti in the diet and ileal chyme, respectively.

Basal endogenous amino acid flow (g/kg)=In ileal digesta amino acid (g/kg) × Td (g/kg) / Ti (g/kg).

Endogenous nitrogen and amino acid values were determined for broiler chickens fed a nitrogen-free diet, and the apparent digestibility data for nitrogen and amino acids were converted to standardized digestibility values.

$$\begin{array}{l} \mathrm{Standardized}\;\mathrm{ileal}\;\mathrm{digestibility}\;(\mathrm{SID},\;\%)=\\ {}[\mathrm{AID}+\mathrm{Basal}\;\mathrm{endogenous}\;\mathrm{amino}\;\mathrm{acid}\;\mathrm{flow}\;(g/\mathrm{kg}\;\mathrm{DMI})]\;/\;\mathrm{In}\;\mathrm{ileal}\;\mathrm{digesta}\;\mathrm{amino}\;\mathrm{acid}\;(\mathrm{Ing}\;\mathrm{AA},\;g/\mathrm{kg}\;\mathrm{DM}), \end{array}$$

The ileal apparent digestibility, the basic endogenous AA loss, and the AA concentration in the ileal digesta are represented by the variables AID, Basal EAA, and Ing AA.

### 2.3. Animals and Diets

The study involved 180 Arbor Acres (AA) broiler chickens weighing 42.43 ± 0.45 g. The chickens were randomly divided into three treatments, each with six replicates and 10 broilers per replicate. The experiment lasted for 42 days and was divided into two feeding phases. There were two phases: the starter phase, which started on day one and ended on day 21, and the grower phase, which started on day 22 and ended on day 42. The control group (CON group) was provided with a basal diet formulated to meet the standards set by the National Research Council (1994) and the Ministry of Agriculture of the People’s Republic of China (2004). The treatment groups consisted of chickens fed the basal diet with 25% (CPC-1) or 50% (CPC-2) of soybean meal replaced with CPC. The ingredients and nutrient content of the experimental diets were shown in Table 3. The chemical composition, amino acid composition, and metabolizable and net energy values of CPC sample were shown in Table 4. The broilers were arranged in three layers of metal cages (140 cm × 70 cm × 40 cm). Broilers were raised in environmentally controlled facilities with fiberglass feeders and plastic grid floors, with feed and purified water provided. The broiler chickens were exposed to 16 h of light and 8 h of darkness. The chicken coops were heated using water circulation heating. The temperature was initially set at 33 °C for the first 3 days and then gradually decreased by 2 °C every week until it reached a steady temperature of 24 °C. Broiler feeding management was in accordance with the Arbor Acre broiler management guidelines, and they were inoculated with Newcastle disease virus vaccine and inactivated infectious bursal disease vaccine according to routine procedures.

### 2.4. Performance Parameters

There were two nutrition stages in the experiment: the starter stage (1–21 days) and the grower stage (22–42 days). The broilers’ average daily gain (ADG) and average daily feed intake (ADFI) were measured during these stages, as well as during the entire experiment period (1–42 days). The feed-to-gain (F/G) was calculated, and the number of broiler deaths was recorded and weighed daily to correct the F/G.

### 2.5. Blood Sample Collection and Analysis

At the age of 42 days, two broilers with comparable weights were chosen from each replicate after a 12 h fast for blood collection from the brachial wing vein. Around 5 mL of blood was collected in an EDTA tube and stored at a temperature of −20 °C for hematological analysis. Fully automatic blood cell analyzer (BC-5000, Mindray Biomedical Electronics Co., Ltd., Guangzhou, China) was used to determine the white blood cell count (WBC), lymphocyte absolute value (LYM), intermediate cell absolute value (MID), granulocyte absolute value (GRA), red blood cell count (RBC), hemoglobin (Hb), hematocrit (HCT), platelet count (PLT), and platelet count (PCT) in the blood.

To collect serum, the blood samples were centrifuged at 3000× *g* and 4 °C for 15 min. The collected serum was stored at −80 °C for future analysis of serum biochemical parameters, antioxidant parameters, and immunoglobulin parameters. The contents of total protein (TP), albumin (ALB), globulin (GLB), alkaline phosphatase (ALP), alanine aminotransferase (ALT), lactate dehydrogenase (LDH), aspartate aminotransferase (AST), lipoprotein (LP), triglyceride (TG), total cholesterol (T-CHO), and blood urea nitrogen (BUN) in serum were determined using an automatic biochemical analyzer (AU5800, American Beckman Coulter Co., Ltd., Washington, DC, USA).

Serum samples were used to assess serum antioxidant capacity as well as immunoglobulin content. The reagent kit’s operating instructions (Nanjing Jiancheng Biotechnology Co., Ltd., Nanjing, China) were followed to measure the serum total antioxidant capacity (T-AOC, A015-1-2, Colorimetric method), superoxide dismutase (SOD, A001-3-2, WST-1 method), glutathione peroxidation (GSH-Px, A005-1-2, Colorimetric method), and malondialdehyde (MDA, A003-1-1, TBA method), as well as immunoglobulin A (IgA, H108-1-2), immunoglobulin M (IgM, H109-1-2), and immunoglobulin G (IgG, H106-1-1).

### 2.6. Statistical Analysis

The Shapiro–Wilk and Levene’s tests were used to verify the normal distribution and homogeneity of variances of the data. The experiment data were analyzed using one-way ANOVA and the General Linear Model (GLM) in SAS 9.4 (SAS Institute Inc., Cary, NC, USA). Variations among the treatments were compared using Tukey’s multiple range tests. Results were presented as the mean and standard error of the mean (SEM). All statements of significance are based on a probability of *p* < 0.05.

## 3. Results

### 3.1. Digestibility of CPC in the Broilers’ Terminal Ileum

The DM and protein digestibility of CPC in the broiler ileum are presented in Table 5. The DM digestibility of CPC in the broiler ileum was found to be 75.61%, while the apparent and standardized digestibility of the protein were 77.71% and 77.90%, respectively. Table 6 demonstrates that the digestibility of Lys, Met, and Thr exceeded 60%, while His, Iso, Leu, Val, Ala, Gly, Pro, Ser, Cys, and Asp exceeded 70%. Additionally, the ileal digestibility of Glu and Arg surpassed 80% and 90%, respectively.

### 3.2. Growth Performance

Table 7 illustrates the impact of replacing soybean meal with CPC on the growth performance of broiler chickens. During the starter phase (1–21 days), the ADG of the CPC-2 group was lower compared to the CON group (*p* < 0.05), but not significantly different from the CPC-1 group (*p* > 0.05). The ADFI of the CON and CPC-1 groups did not show significant differences (*p* > 0.05), but both groups had a higher ADFI compared to the CPC-2 group (*p* < 0.05). Furthermore, the F/G ratio increased in the CPC-1 and CPC-2 groups compared to the CON group (*p* < 0.05), but there were no significant differences between the CPC-1 and CPC-2 groups (*p* > 0.05). In the grower phase (22–42 d), the ADG of the CON and CPC-1 groups were higher than the CPC-2 group (*p* < 0.05). The inclusion of CPC resulted in a significant decrease in the ADFI of broilers in both the CPC-1 and CPC-2 groups (*p* < 0.05), and the CPC-2 group was significantly lower than that of the CPC-1 group (*p* < 0.05). The F/G of the CPC-1 and CPC-2 groups were significantly decreased compared to the CON group (*p* < 0.05). Throughout the entire duration of the experiment (1–42 d), there were no significant differences in FBW between the CON and CPC-1 groups (*p* > 0.05), although both groups exhibited higher FBW compared to the CPC-2 group (*p* < 0.05). The FI of the CPC-2 group was significantly lower compared to the CON and CPC-1 groups (*p* < 0.05), and the CON group had a higher FI than the CPC-1 group (*p* < 0.05). The CON and CPC-1 groups were not significantly different in ADG (*p* > 0.05), but higher than the CPC-2 group (*p* < 0.05). The same trend as the grower phase, the ADFI decreased sequentially in the CON, CPC-1, and CPC-2 groups (*p* < 0.05). Furthermore, the CPC-1 group was reduced in F/G compared to the CON and CPC-2 groups (*p* < 0.05).

### 3.3. Blood Parameters

Table 8 presents the impact of CPC substitute for soybean meal in the diet on the hematological parameters of broiler chickens. The results showed no significant alternations in blood WBC, LYM, MID, GRA, RBC, HGB, HCT, PLT, and PCT parameters in the CON, CPC-1, and CPC-2 groups (*p* > 0.05).

### 3.4. Serum Biochemistry

Table 9 presents the impact of substituting CPC for soybean meal in the diet on the serum biochemical indices of broiler chickens. The ALP and ALT parameters in the CPC-1 and CPC-2 groups were not significantly altered (*p* > 0.05), but higher than the CON group (*p* < 0.05). No statistically significant differences were observed for the TP, ALB, GLB, LDH, AST, LP, TG, TCHO, and BUN between the CON, CPC-1, and CPC-2 groups (*p* > 0.05).

### 3.5. Serum Antioxidant Capacity

Results of the serum antioxidant capacity parameters were presented in Table 10. The T-AOC and SOD antioxidant enzyme activity in the CON and CPC-2 groups were not significantly altered (*p* > 0.05), but lower than the CPC-1 group (*p* < 0.05). The MDA content was found to be significantly lower in the CPC-1 group compared to the CON and CPC-2 groups (*p* < 0.05), and there was no significant difference observed between the CON and CPC-2 groups (*p* > 0.05). However, no significant difference in serum GSH-Px activity was observed between the CON, CPC-1, and CPC-2 groups (*p* > 0.05).

### 3.6. Serum Immunoglobulin Content

The serum immunoglobulin concentrations of the broiler chickens were summarized in Table 11. The IgA and IgM levels of the CON and CPC-2 groups did not exhibit a significant difference (*p* > 0.05). However, both groups displayed significantly lower levels compared to the CPC-1 group (*p* < 0.05). On the other hand, the IgG content was found to be significantly higher in both the CPC-1 and CPC-2 groups as compared to the CON group (*p* < 0.05), and the CPC-2 group was also higher than that of the CPC-1 group (*p* < 0.05).

## 4. Discussion

Fish generally have a significant tolerance to free gossypol in their diet, making CPC a viable non-grain protein source for aquaculture [14]. Currently, CPC experiments are primarily assessing whether it can replace some fish meal to maintain or improve the growth performance of aquatic species. Yin et al. found that CPC can significantly improve the production performance of aquaculture by replacing fish meal [9]. He et al. found that feed containing 5.76% and 18.18% CPC levels promoted the growth performance of hybrid grouper [15]. Additionally, Liu et al. observed that replacing 25% and 50% of fish meal with CPC in the diet had no significant effects on the growth performance of largemouth bass. However, when 75% of CPC was used as a dietary substitution, it resulted in a significant decrease in FBW, WGR, SGR, and feed efficiency [16]. The observed variations could be attributed to various factors such as the type of fish, their growth stages, the basic feed formulas used, and the differing feeding environments. In summary, the above results confirm the potential of CPC as a protein raw material to replace conventional protein raw materials in feed. The viability of using CPC with broilers is further supported by this analysis of ileal digestibility, energy content, and chemical composition. CPC considerably raises the amount of sulfur-containing amino acids (methionine + cysteine), and other essential amino acids like arginine and lysine, when compared to soybean meal. Additionally, CPC’s protein and amino acid digestibility are both above 60%, meaning that broilers effectively utilize its amino acid content. Therefore, we attempted to use CPC as a substitute for different levels of soybean meal in the diet to observe the impact on the growth and development of broilers. This study discovered that substituting 50% of soybean meal in the diet with CPC had significant negative impacts on early growing broilers, resulting in reduced ADG and ADFI. Meanwhile, the growth and development of broilers were affected by the free gossypol content in diets that have high CPC content, even after undergoing dephenolization treatment [17,18]. This result indicates that replacing 50% of soybean meal in broiler feed with CPC has a significant negative impact on production performance, which should not be overlooked. In contrast, substituting CPC for 25% soybean meal in the broilers’ diet resulted in a reduction in ADFI and F/G, without compromising their normal growth and development. This finding suggests that the application of CPC may be feasible.

The above results were also confirmed through blood parameters. The activity levels of ALP, AST, and ALT are commonly used as diagnostic tools to assess hepatotoxicity [19,20]. Elevated levels of ALT and AST often indicate liver damage and impaired liver function [21,22]. Liu et al. observed a linear increase in the serum ALT index as the proportion of fish meal in the CPC substitute diet increased [8]. In this study, substituting 25% and 50% soybean meal in broiler diets with CPC did not result in significant changes in blood routine indicators. However, the CPC-1 and CPC-2 groups showed noticeable increases in serum ALP and ALT indicators compared to the CON group. This result indicates that feeding a diet containing CPC may had adverse effects on liver health. The toxicity of free gossypol contained in CPC was primarily attributed to the aldehyde and hydroxyl groups within its structure [23]. This compound can accumulate within an animal liver and bind with metal ions and amino acid residues, resulting in damage to its original biological function and ultimately causing harm to liver function [24]. ALP is an enzyme found in the liver bile ducts [25]. The damage of the liver often results in the damage of the intrahepatic bile ducts, which then affects the normal metabolism of ALP in the liver [26]. The results indicate that, although CPC exhibits the potential to replace soybean meal in broiler diets, its negative effects still need to be carefully considered.

The imbalance between antioxidants and free radicals leads to oxidative stress, which will produce reactive oxygen species (ROS) [27,28]. Excessive ROS increases the content of MDA, thus causing damage to tissues [29]. T-AOC, SOD, and GSH-Px are considered essential markers of antioxidant capacity, which eliminates excessive ROS in the body [30,31]. Liu et al. found that the T-AOC and activity of GSH-Px in the hepatopancreas of grass carp fed with CPC significantly increased, while the content of MDA significantly decreased [7]. This study found that the serum activities of T-AOC and SOD in broilers from the CPC-1 group were significantly increased, while the content of MDA was significantly reduced. This could be attributed to the high amino acid digestibility of the CPC diet, which improved the overall development of broilers and consequently enhanced their antioxidant capacity. In addition, IgA, IgG, and IgM are considered important parameters reflecting the humoral immune status of animals [32,33]. The level of serum immunoglobulin to a certain extent reflects the immune function of animals, which helps to alleviate immune stress, improve health status, and improve growth performance [34]. However, the increase in immunoglobulin levels may also be influenced by other factors, such as the body’s immune response to antigens. The study observed that the serum levels of IgA, IgM, and IgG were significantly higher in the CPC-1 group compared to the CON group. However, there was no significant difference in the levels of IgA and IgM between the CON and CPC-2 groups. The results mentioned above suggest that there may not be a significant correlation between the increase in immunoglobulin content in the CPC-1 group and the body’s antigen response. It was possible, nevertheless, that the CPC-2 group’s elevated IgG content resulted from an antigen reaction triggered by gossypol. It is important to note that these results are speculative and further research is necessary to investigate the factors contributing to the changes in immune function in broilers.

## 5. Conclusions

This study demonstrates that replacing 25% soybean meal with CPC in the diet of broilers leads to a significant reduction in ADFI and F/G, while maintaining normal growth and development. Furthermore, broilers in the CPC-1 group exhibited better antioxidant capacity and immune function. However, substituting 50% soybean meal for a CPC diet had a significant negative effect, as is reflected in the decrease in ADG and the increase in serum ALT and ALP. While CPC has the potential to serve as a viable alternative to soybean meal in the broiler diet, it is important to consider the negative effects associated with the amount of CPC supplemented in the diet. More research is needed to thoroughly investigate blood indicators and understand the importance of using CPC in broiler diets. This research will aid in determining the proper dosage for safe and effective use.

## Figures and Tables

**Table 1:** The composition of CPC diet for apparent metabolizable energy and net energy experiment in broiler chickens (%, as-is basis).

Items	Basal Diet	CPC Diet
Corn	58.42	46.74
46% Soybean meal	26.12	20.02
93% Casein	6.48	5.18
Soybean oil	4.60	3.68
62.98% CPC	-	20.00
Limestone	1.38	1.38
CaHPO_4_	2.11	2.11
NaCl	0.28	0.28
Choline chloride	0.08	0.08
*DL*-Methionine	0.03	0.03
Premix ^1^	0.13	0.13
TiO_2_	0.37	0.37
Total	100.00	100.00
Chemical composition ^2^		
Metabolizable energy, MJ/kg	13.17	13.03
Crude protein	22.00	28.13
Calcium	1.00	0.99
Phosphorus	0.46	0.47

^1^ The premix provided the following per kilogram diet: vitamin A 12, 500 IU, vitamin D_3_ 3500 IU, vitamin E 20 IU, vitamin K_3_ 3 mg, vitamin B_1_ 0.01 mg, vitamin B_2_ 8 mg, vitamin B_3_ 10 mg, vitamin B_5_ 40 mg, vitamin B_6_ 3 mg, vitamin B_11_ 0.3 mg, vitamin B_12_ 0.03 mg, biotin 0.2 mg, Cu (as copper sulfate) 8 mg, Fe (as ferrous sulfate) 80 mg, Mn (as manganese sulfate) 80 mg, Zn (as zinc sulfate) 80 mg, Se (as sodium selenite) 0.3 mg, I (as potassium iodide) 0.7 mg. ^2^ Calculated chemical composition concentrations.

**Table 2:** The composition of experiment diets for the digestibility rate of dry matter, protein, and amino acid of CPC in the ileum of broiler chickens (%, as-is basis).

Items	Nitrogen-Free Diet	CPC Diet
Saccharose	19.97	16.00
Corn starch	68.10	40.76
Avicel	5.00	-
Soybean oil	3.00	-
62.98% CPC	-	39.61
Limestone	1.00	0.94
CaHPO_4_	1.90	1.66
NaCl	0.30	0.30
Choline chloride	0.10	0.10
Premix ^1^	0.13	0.13
Zeolite	0.10	0.10
TiO_2_	0.40	0.40
Total	100.00	100.00
Chemical composition ^2^		
Metabolizable energy, MJ/kg	13.31	11.99
Crude protein	0.45	25.95
Calcium	0.92	1.00
Phosphorus	0.43	0.48

^1^ The premix provided the following per kilogram diet: vitamin A 12, 500 IU, vitamin D_3_ 3500 IU, vitamin E 20 IU, vitamin K_3_ 3 mg, vitamin B_1_ 0.01 mg, vitamin B_2_ 8 mg, vitamin B_3_ 10 mg, vitamin B_5_ 40 mg, vitamin B_6_ 3 mg, vitamin B_11_ 0.3 mg, vitamin B_12_ 0.03 mg, biotin 0.2 mg, Cu (as copper sulfate) 8 mg, Fe (as ferrous sulfate) 80 mg, Mn (as manganese sulfate) 80 mg, Zn (as zinc sulfate) 80 mg, Se (as sodium selenite) 0.3 mg, I (as potassium iodide) 0.7 mg. ^2^ Calculated chemical composition concentrations.

**Table 3:** Ingredients and nutrient content of experimental diets (%, as-is basis).

Items	Contents					
	1–21 d			22–42 d		
	CON	CPC-1	CPC-2	CON	CPC-1	CPC-2
Corn	55.18	59.11	61.92	58.02	61.28	64.54
46% Soybean meal	30.26	23.31	16.66	23.98	17.64	11.28
62.98% CPC	0.00	4.00	8.00	0.00	4.00	8.00
Soybean oil	2.06	0.85	0.00	3.08	1.98	0.87
49.42% Peanut meal	3.00	3.00	3.00	3.00	3.00	3.00
27% DDGS	5.00	5.00	5.00	8.00	8.00	8.00
NaCl	0.32	0.32	0.32	0.32	0.32	0.32
CaHPO_4_	1.73	1.77	1.83	1.44	1.49	1.53
Limestone	1.41	1.45	1.97	1.20	1.23	1.26
*L*-Lysine	0.39	0.47	0.55	0.37	0.45	0.52
*DL*-Methionine	0.32	0.32	0.32	0.28	0.28	0.28
*L*-Threonine	0.00	0.06	0.09	0.02	0.06	0.11
*L*-Tryptophan	0.00	0.02	0.01	0.00	0.00	0.01
Choline chloride	0.20	0.20	0.20	0.15	0.15	0.15
Premix ^1^	0.13	0.13	0.13	0.13	0.13	0.13
Chemical composition ^2^						
Metabolizable energy, MJ/kg	12.55	12.55	12.55	12.97	12.97	12.97
Crude protein	20.50	20.50	20.50	18.50	18.50	18.50
Calcium	1.000	1.000	1.176	0.850	0.850	0.850
Phosphorus	0.679	0.652	0.626	0.624	0.600	0.575
Nonphytate phosphorus	0.450	0.450	0.450	0.420	0.420	0.420
Lysine	1.250	1.250	1.250	1.100	1.100	1.100
Methionine	0.632	0.628	0.625	0.566	0.563	0.560
Threonine	0.800	0.809	0.800	0.720	0.720	0.720
Methionine + Cysteine	0.950	0.950	0.950	0.850	0.850	0.850
Tryptophan	0.260	0.263	0.240	0.221	0.208	0.200

^1^ The premix provided the following per kilogram diet: (1–21 d) vitamin A 10,000 IU, vitamin D_3_ 2000 IU, vitamin E 10 IU, vitamin K_3_ 2.5 mg, vitamin B_1_ 1.8 mg, vitamin B_2_ 4 mg, vitamin B_3_ 50 mg, vitamin B_5_ 11 mg, vitamin B_9_ 0.5 mg, vitamin B_12_ 0.7 mg, biotin 0.12 mg, Cu (as copper sulfate) 8 mg, Fe (as ferrous sulfate) 80 mg, Mn (as manganese sulfate) 60 mg, Zn (as zinc sulfate) 80 mg, Se (as sodium selenite) 0.15 mg, I (as potassium iodide) 0.35 mg. (22–42 d) vitamin A 8000 IU, vitamin D_3_ 1500 IU, vitamin E 8 IU, vitamin K_3_ 2.0 mg, vitamin B_1_ 1.5 mg, vitamin B_2_ 3 mg, vitamin B_3_ 40 mg, vitamin B_5_ 9 mg, vitamin B_9_ 0.4 mg, vitamin B_12_ 0.6 mg, biotin 0.10 mg, Cu (as copper sulfate) 6 mg, Fe (as ferrous sulfate) 60 mg, Mn (as manganese sulfate) 50 mg, Zn (as zinc sulfate) 60 mg, Se (as sodium selenite) 0.12 mg, I (as potassium iodide) 0.30 mg. ^2^ The numerical value represents the calculated content of chemical composition.

**Table 4:** The chemical composition of CPC (as-is basis).

Items ^1^	CPC
Chemical composition (*n* = 6)	
DM, %	92.80
CP, %	62.98
EE, %	0.53
CF, %	4.53
Ash, %	7.89
Indispensable amino acids (*n* = 6)	
Lys, %	2.45
Met, %	0.81
Arg, %	7.74
Thr, %	1.86
His, %	1.79
Val, %	2.56
Leu, %	3.52
Iso, %	1.90
Phe, %	3.32
Dispensable amino acids (*n* = 6)	
Gly, %	2.40
Ser, %	2.65
Pro, %	2.20
Ala, %	2.29
Asp, %	5.70
Glu, %	12.79
Cys, %	1.07
Energy values (*n* = 6)	
AME, kJ/g	11.76
NE, kJ/g	6.11

^1^ CPC, cottonseed protein concentrate. DM, Dry matter; CP, crude protein; EE, ether extract; CF, crude fibre; Lys, lysine; Met, methionine; Arg, arginine; Thr, threonine; His, histidine; Val, valine; Leu, leucine; Iso, isoleucine; Phe, phenylalanine; Gly, glycine; Ser, serine; Pro, Proline; Ala, alanine; Asp, aspartic acid; Glu, glutamic; Cys, cystine. AME, Apparent metabolizable energy; NE, Net energy.

**Table 5:** Ileal digestibility of dry matter and protein in broiler chickens for CPC (%).

Items	CPC
Apparent ileal digestibility of dry matter (*n* = 6)	
Dry matter, %	75.61
Apparent ileal digestibility of protein (*n* = 6)	
Crude protein, %	77.71
Standardized ileal digestibility of protein (*n* = 6)	
Crude protein, %	77.90

**Table 6:** The amino acid apparent and standardized digestibility of CPC in the broilers’ ileum (%).

Items ^1^	Ileal Amino Acid Digestibility	
	Apparent	Standardized
Apparent ileal digestibility of amino acid (*n* = 6)		
Lys	69.34	69.38
Met	68.89	68.95
Arg	90.47	90.32
His	76.69	76.76
Iso	72.65	72.72
Leu	76.10	76.16
Thr	63.77	63.92
Val	75.05	75.13
Ala	71.49	71.56
Gly	73.90	73.97
Glu	87.61	87.63
Pro	76.23	76.33
Ser	74.36	75.45
Cys	72.69	72.76
Asp	75.06	76.59

^1^ Lys, lysine; Met, methionine; Arg, arginine; His, histidine; Iso, isoleucine; Leu, leucine; Thr, threonine; Val, valine; Ala, alanine; Gly, glycine; Glu, Glutamic acid; Pro, proline; Ser, serine; Cys, cysteine; Asp, aspartic acid.

**Table 7:** Effects of CPC substitute for soybean meal in the diet on growth performance of broiler chickens.

Items	CON	CPC-1	CPC-2	SEM	*p*-Value
Starter phase (1–21 d)					
ADG, g	31.99 ^a^	29.17 ^ab^	26.69 ^b^	0.783	0.009
ADFI, g/d	46.89 ^a^	45.18 ^a^	40.99 ^b^	0.839	0.003
F/G	1.47 ^b^	1.55 ^a^	1.54 ^a^	0.021	0.023
Grower phase (22–42 d)					
ADG, g	68.85 ^a^	69.03 ^a^	65.69 ^b^	0.712	<0.001
ADFI, g/d	99.57 ^a^	96.15 ^b^	92.05 ^c^	3.335	<0.001
F/G	1.45 ^a^	1.40 ^b^	1.40 ^b^	0.021	0.012
Overall phase (1–42 d)					
IBW, g	42.35	42.47	42.29	0.936	0.847
FBW, kg	2.16 ^a^	2.15 ^a^	1.99 ^b^	0.038	<0.001
FI, kg	30.88 ^a^	29.69 ^b^	27.96 ^c^	1.093	<0.001
ADG, g	50.42 ^a^	49.95 ^a^	46.06 ^b^	0.554	<0.001
ADFI, g/d	73.23 ^a^	70.68 ^b^	66.51 ^c^	1.367	<0.001
F/G	1.46 ^a^	1.42 ^b^	1.45 ^a^	0.015	<0.001

*n* = 6. ADG, average daily gain; ADFI, average daily feed intake; F/G, feed-to-gain; IBW, initial body weight; FBW, final body weight; FI, feed intake. CON group, basal diet in control group; CPC-1 group, cottonseed protein concentrate replaces 25% soybean meal in the diets; CPC-2 group, cottonseed protein concentrate replaces 50% soybean meal in the diets. ^a–c^ Means within a row lacking a common superscript differ (*p* < 0.05).

**Table 8:** Effects of CPC substitute for soybean meal in the diet on hematological parameters of broiler chickens.

Items	CON	CPC-1	CPC-2	SEM	*p*-Value
WBC, 10^9^/L	111.22	107.71	113.79	1.972	0.243
LYM, 10^9^/L	63.57	63.26	64.10	0.563	0.844
MID, 10^9^/L	15.84	14.67	16.05	0.285	0.099
GRA, 10^9^/L	31.81	30.78	32.64	2.069	0.524
RBC, 10^12^/L	2.59	2.64	2.64	0.061	0.940
HGB, g/L	98.33	99.67	99.27	2.365	0.564
HCT, L/L	0.23	0.24	0.26	0.006	0.246
PLT, 10^9^/L	14.47	15.50	15.19	1.594	0.183
PCT, L/L	0.02	0.02	0.01	0.002	0.265

*n* = 6. WBC, white blood cell count; LYM, lymphocyte absolute value; MID, intermediate cell absolute value; GRA, granulocyte absolute value; RBC, red blood cell count; HGB, hemoglobin; HCT, hematocrit; PLT, platelet count; PCT, platelet count. CON group, basal diet in control group; CPC-1 group, cottonseed protein concentrate replaces 25% soybean meal; CPC-2 group, cottonseed protein concentrate replaces 50% soybean meal.

**Table 9:** Effects of CPC substitute for soybean meal in the diet on serum biochemical indices of broiler chickens.

Items	CON	CPC-1	CPC-2	SEM	*p*-Value
TP, mg/mL	110.04	109.59	110.73	3.813	0.559
ALB, mg/mL	38.89	38.96	37.79	1.504	0.394
ALP, ng/mL	122.10 ^b^	149.53 ^a^	147.96 ^a^	5.514	<0.001
GLB, g/L	21.25	22.41	22.03	1.431	0.114
LDH, ng/mL	10.92	13.06	11.89	0.566	0.319
ALT, mmoL/L	144.71 ^b^	210.93 ^a^	212.67 ^a^	11.249	0.003
AST, ng/mL	272.82	269.34	277.60	12.728	0.706
LP, μg/mL	287.78	278.80	283.29	11.570	0.302
TG, mg/mL	0.662	0.663	0.623	0.030	0.846
TCHO, μmol/dL	421.38	427.88	412.36	16.959	0.194
BUN, mg/mL	0.21	0.20	0.20	0.011	0.609

*n* = 6. TP, total protein; ALB, albumin; GLB, globulin; ALP, alkaline phosphatase; ALT, alanine aminotransferase; LDH, lactate dehydrogenase; AST, aspartate aminotransferase; LP, lipoprotein; TG, triglyceride; TCHO, total cholesterol; BUN, blood urea nitrogen. CON group, basal diet in control group; CPC-1 group, cottonseed protein concentrate replaces 25% soybean meal; CPC-2 group, cottonseed protein concentrate replaces 50% soybean meal. ^a,b^ Means within a row lacking a common superscript differ (*p* < 0.05).

**Table 10:** Effects of CPC substitute for soybean meal in the diet on serum antioxidant capacity parameters of broiler chickens.

Items	CON	CPC-1	CPC-2	SEM	*p*-Value
T-AOC, U/mL	0.17 ^b^	0.22 ^a^	0.18 ^b^	0.007	0.031
SOD, U/mL	218.94 ^b^	369.62 ^a^	249.82 ^b^	20.064	0.002
MDA, nmoL/mL	12.28 ^a^	8.41 ^b^	11.26 ^a^	0.464	<0.001
GSH-Px, U/mL	147.01	146.93	141.35	4.314	0.845

*n* = 6. T-AOC, total antioxidant capacity; SOD, superoxide dismutase; MDA, Malondialdehyde; GSH-Px, Glutathione peroxidase. CON group, basal diet in control group; CPC-1 group, cottonseed protein concentrate replaces 25% soybean meal; CPC-2 group, cottonseed protein concentrate replaces 50% soybean meal. ^a,b^ Means within a row lacking a common superscript differ (*p* < 0.05).

**Table 11:** Effects of CPC substitute for soybean meal in the diet on serum immunoglobulin concentrations of broiler chickens.

Item	CON	CPC-1	CPC-2	SEM	*p*-Value
IgA, μg/mL	116.21 ^b^	221.90 ^a^	156.08 ^b^	13.147	<0.001
IgM, μg/mL	552.84 ^b^	731.41 ^a^	624.14 ^b^	25.733	0.008
IgG, μg/mL	1328.03 ^c^	1450.93 ^b^	1769.05 ^a^	66.852	<0.001

*n* = 6. IgA, immunoglobulin A; IgM, immunoglobulin M; IgG, immunoglobulin G. CON group, basal diet in control group; CPC-1 group, cottonseed protein concentrate replaces 25% soybean meal; CPC-2 group, cottonseed protein concentrate replaces 50% soybean meal. ^a–c^ Means within a row lacking a common superscript differ (*p* < 0.05).

## Data Availability

The datasets used and analyzed during the current study are available from the corresponding author on reasonable request.
